# Utilizing Chamber Music to Teach Non-Verbal Communication to Medical Students: A Pilot Initiative

**DOI:** 10.7759/cureus.14587

**Published:** 2021-04-20

**Authors:** Leo M Hall, Connor Buechler, Georgiana Marusca, Simone Brennan, Diane L Levine

**Affiliations:** 1 Internal Medicine, Wayne State University School of Medicine, Detroit, USA; 2 Office of Learning and Teaching, Wayne State University School of Medicine, Detroit, USA; 3 Internal Medicine, Wayne State University Detroit Medical Center, Detroit, USA

**Keywords:** medical education, interpersonal and communication skills, nonverbal communication

## Abstract

Introduction

The importance of non-verbal cues in communication between physicians and patients is well published in the medical literature. However, few medical school curricula teach non-verbal communication. Chamber musicians employ non-verbal communication to coordinate musician intention. Observation of chamber musicians’ use of non-verbal communication may improve the understanding of non-verbal communication among medical students.

Methods

A total of 72 medical students attended rehearsals of two world-renowned string quartets on a single date. Following a brief discussion and demonstration on non-verbal communication by musicians, students observed the non-verbal cues employed by the quartets during musical rehearsals. Authors provided pre- and post-surveys, which included closed and open-ended questions to assess understanding of non-verbal communication and confidence in identifying non-verbal cues with patients and healthcare providers. Close-ended questions used numerical scales. The authors used paired t-tests to compare mean numerical scores pre- and post-intervention and analyzed qualitative, open-ended responses thematically.

Results

Of the 72 students who attended the workshop, 63 (88%) completed both pre- and post-surveys. Comparison demonstrated significant improvement in students’ ability to appreciate non-verbal interactions among healthcare teams (p<0.05) and patients (p<0.05). Following the workshop, students commented that they appreciated the similarities in non-verbal cues between musicians and medical professionals.

Discussion

Chamber musicians and physicians share similarities, e.g., working in teams and performing specialized tasks; good communication is crucial to both. Observation of chamber musicians may serve as a vehicle to instruct medical students on non-verbal communication. Next steps include determining the longer-term impact of the workshop on confidence in communication by resurveying participants and comparing responses with those students who did not attend the workshop. Future studies are needed to assess the clinical impact of chamber music observation on medical students’ non-verbal communication skills.

## Introduction

Communication is a critical healthcare competency, and it is essential to high-quality, patient-centered care. Non-verbal communication refers to communication using non-linguistic behaviors [1]. Within the medical literature, the importance of non-verbal communication is well published [2]. Improved non-verbal communication is linked to greater patient satisfaction and compliance [3]. Effective use of non-verbal cues is also essential in fostering effective collaboration and greater efficiencies between colleagues [4].

The humanities have been used to teach medical students communication, including improvisational communication [5] and non-verbal communication [6]. Musicians have been shown to exhibit a greater sensitivity to detecting speech amid noise as compared to non-musicians [7], which is advantageous when in environments in which competing sound interests complicate communication. Chamber musicians rely on non-verbal communication to synchronize their musical intentions without a conductor. Observation of chamber musicians in practice may therefore serve as a conduit through which non-verbal communication cues may become well-digestible to medical students. Similar studies involving jazz music have focused on the importance of improvisation and lessening barriers to pedagogical norms that influence instructing on communication [5]; however, no studies have focused in particular on non-verbal communication nor have they investigated the impact of a compressed workshop curriculum involving classical musicians on communication.

We invited two world-renowned string quartets, the Thalea and Emerson String Quartets, to provide a workshop, which included observed rehearsals, on non-verbal communication. We developed a survey to capture (1) baseline knowledge regarding non-verbal communication skills used by musicians and (2) students’ confidence in identifying non-verbal cues with patients and other health professionals. Following the workshop, we resurveyed students to assess (1) knowledge and observations gained and (2) students’ confidence in identifying non-verbal cues with patients and other health professionals. We also investigated how race and prior musical experience affected participants’ confidence in recognizing non-verbal cues. This work was presented at the Northeast Group on Educational Affairs (NEGEA) Health Humanities Special Interest Group (SIG) Pre-Conference Symposium on May 19, 2020.

## Materials and methods

A total of 72 Wayne State University School of Medicine third-year medical students on their Internal Medicine and Pediatrics clerkships attended a rehearsal of two renowned string quartets, the Thalea and Emerson String Quartets. This workshop occurred at the beginning of the clinical year, specifically during the first clinical rotation for all participants. Prior to the workshop, students were provided pre-reading material, through which they were introduced to non-verbal cues and their relevance in patient-physician relations. To begin the workshop, chamber musicians introduced the topic of non-verbal communication and described how they employed non-verbal cues as a means of synchronizing musical intent. Descriptions of different techniques and tools were followed by short musical demonstrations. Students then observed the quartets rehearsing musical pieces, which lasted approximately five minutes per demonstration. During these observations, students received unique observation task sheets. Observation task sheets were designed to help focus observations on specific non-verbal communication cues and served as the catalyst for small and large-group discussion and debrief. The observation task sheets contained information about (1) kinesic non-verbal cues, e.g., body posture, body gestures, facial expressions, eye contact, and (2) paralinguistic cues, e.g., pitch, volume, rate, quality, and verbal fillers (“um”, “uh”, “mmm”, “uh-huh”, intentional clearing of throat, etc.). In addition to noting the specific actions performed by the quartets, students were also asked to note the frequency with which these actions occurred. An example page from the observation task sheet can be found in Appendix A. Following the brief performances, students worked in small groups to discuss the non-verbal cues used by the chamber musicians. These small groups were comprised of four randomly assorted students (student "quartets”); each small group session lasted between 10 and 15 minutes. These sessions were followed by short five-minute debriefings during which faculty members asked probing questions developed to stimulate conversation, including a discussion about the overlap between chamber music and interprofessional communication. Faculty authors served as discussants for the small group debriefs. Discussants received no prior training; however, they were provided a list of questions prior to the session and were asked to review these questions in preparation for the session (Appendix B). Questions were developed thematically and focused on elements that readily compare between chamber musicians and healthcare providers, e.g., communication among teams and iterative practice. One day prior to the session, students received a link to the pre-survey to complete prior to the session. Authors provided a post-survey link at the conclusion of the workshop. Pre- and post-survey questions were formulated to measure changes before and after the workshop (Appendix C). Demographic data, including self-identified gender, race, and ethnicity, were also collected. Students were able to select all races and ethnicities to which they belong. Students were also asked about previous musical experience.

Survey questions were developed inductively and the questions focused to reflect the unique experience of observing chamber musicians. The questions were evaluated using an iterative process in an effort to decrease redundancy and avoid leading, loaded, or double-barreled questions. Quantitative data were captured using a 4-point numerical scale (high confidence, moderate confidence, slight confidence, no confidence) and a 5-point numerical scale (strongly agree, agree, neither agree nor disagree, disagree, strongly disagree). Open-ended, free-response questions were employed to capture qualitative data.

Comparisons between responses on numerical scales were made using unpaired t-tests, with statistical significance determined using the Holm-Sidak method and alpha = 0.05. Each test was analyzed individually without assuming a consistent standard deviation. Statistics were performed using Graphpad Prism software Version 8 (GraphPad Software, Inc., La Jolla, CA).

Qualitative analysis in this study applied a modified version of a phronetic iterative approach identifying emergent themes. Briefly, data were assessed in a stepwise manner, starting with data immersion, followed by development of first- and second-order codes, identification of linkages among answers, and, finally, development of hierarchical code families comprising overarching themes. This analytic approach was chosen for its systematic stepwise qualitative coding, ensuring a measured approach to identifying themes in the data. This manner ensured greater transparency in how the coding was conducted, thereby providing both accountability and replicability. Additionally, documenting findings using stepwise coding demonstrates “rich rigor”. The study was reviewed by the Wayne State University Institutional Review Board and was performed in accordance with the Declaration of Helsinki.

## Results

**Figure 1:**
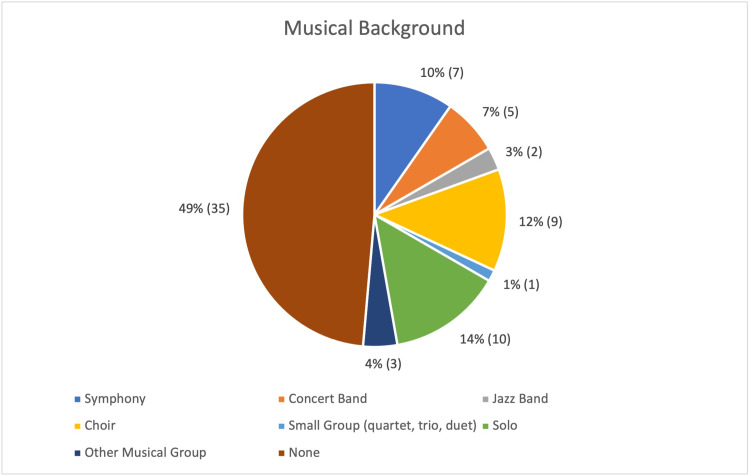
Musical backgrounds of participants. Almost half of participants self-reported no musical background. Of those who self-reported prior musical experience, most reported experience in solo performance, choir, and symphony. Data are presented as percentage of total (%) and number reported (n).

A total of 72 (100%) responses to the pre-survey and 63 (88%) responses to the post-survey were received. Demographic makeup of our student population was 56.9% (41/72) white, 25% (18/72) Asian, 8.3% (6/72) black or African American, 0% (0/72) American Indian or Alaskan Native, and 0% (0/72) Native Hawaiian or other Pacific Islander; 12.5% (9/72) of respondents chose not to answer this question and were excluded from our analyses of racial makeup. Slightly more than half of respondents identified as male (data not shown). Many respondents had no prior musical background (49%, 35/72). Students with musical experience included prior participation in (1) small group ensemble (14%, 10/72), (2) choir (12%, 9/72), and (3) symphony (10%, 7/72). A detailed breakdown of musical backgrounds is available in Figure 1.

**Figure 2:**
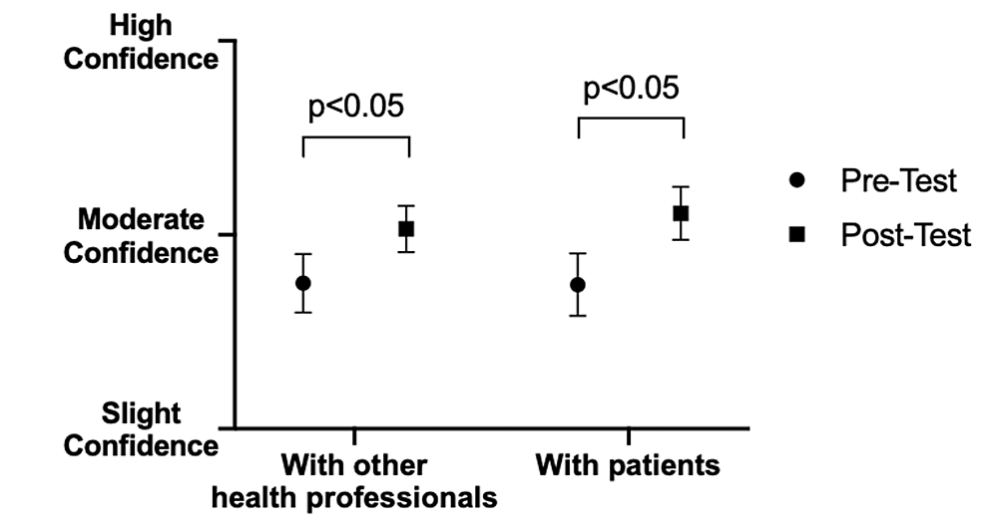
Student confidence identifying non-verbal cues in interactions in pre- and post-intervention. Pre-intervention surveys demonstrated that third-year medical students had less confidence recognizing non-verbal cues both among other health professionals and with patients as compared to post-intervention surveys, which showed significantly improved confidence with respect to both other health professionals and patients (p<0.05).

Student’s self-rated level of confidence in identifying non-verbal cues in interactions with other health professionals improved following intervention (pre: 2.75 vs. post: 3.03; p<0.05), as did their self-rated confidence in identifying such cues in interactions with patients (pre: 2.74 vs. post: 3.11; p<0.05) (Figure 2). Students agreed overall that the knowledge gained from the workshop would help them to identify non-verbal cues in both patients and colleagues and that they would recommend the workshop to other students.

**Figure 3:**
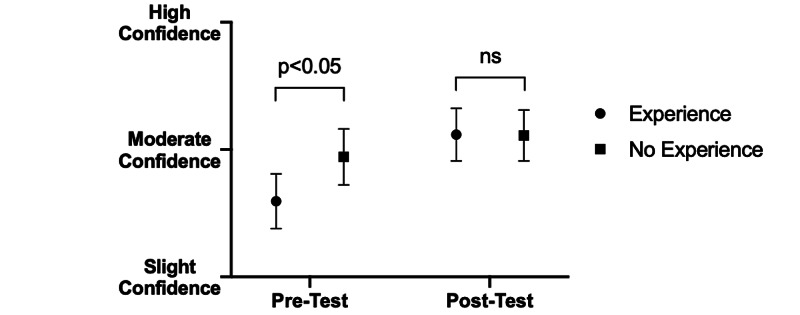
Effect of pre-intervention musical experience on confidence identifying non-verbal cues in patients before and after intervention. Students with past musical experience exhibited significantly less confidence on average in recognizing non-verbal cues in patients among musicians as compared to students without musical backgrounds (p<0.05). Following intervention, this difference in average confidence between groups was removed, and both student cohorts showed improvement in their average confidence in recognizing non-verbal cues.

**Figure 4:**
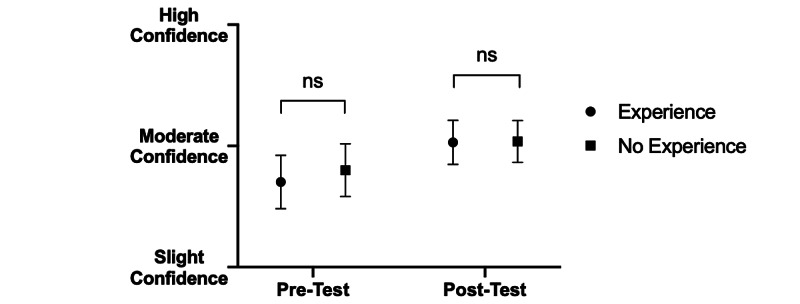
Effect of pre-intervention musical experience on confidence identifying non-verbal cues in healthcare colleagues before and after intervention. When stratified according to musical background, there was no significant difference in average confidence reported by participants pre-intervention in recognizing non-verbal cues among colleagues (p>0.05). Post-intervention yielded increased average confidence in noting non-verbal cues among colleagues in both participants with and without musical experience, although the difference in average confidence was non-significant (p>0.05).

Before the quartet intervention, students with musical backgrounds expressed lower confidence identifying non-verbal cues in patient interactions than did their peers without musical backgrounds (pre: 2.59 vs. post: 2.94; p<0.05) (Figure 3). However, confidence identifying such cues in other healthcare professionals was not affected by prior musical experience (pre: 2.70 vs. post: 2.80: p>0.05) (Figure 4). Following intervention, there were no significant differences in confidence between the two groups in identifying non-verbal cues for interactions with either patients (pre: 3.12 vs. post: 3.11; p>0.05) (Figure 3) or healthcare providers (pre: 3.03 vs. post: 3.04; p>0.05) (Figure 4).

**Figure 5:**
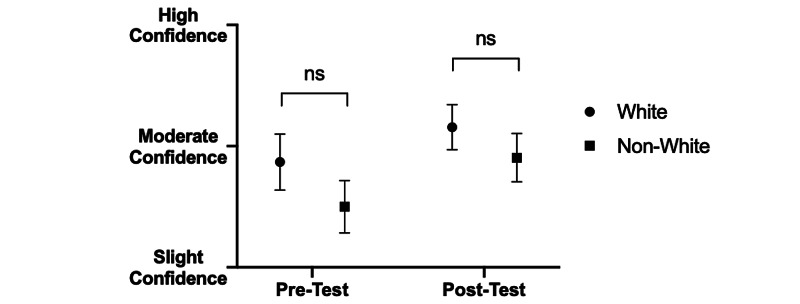
Effect of identifying as white/non-white race on confidence identifying non-verbal cues in healthcare colleagues before and after intervention. White participants on average reported greater confidence in noting non-verbal cues among colleagues in pre-intervention survey as compared to non-white participants, although the difference in average confidence was non-significant (p>0.05). Following intervention, both white and non-white average confidence increased, and the difference in average confidence remained non-significant (p>0.05).

**Figure 6:**
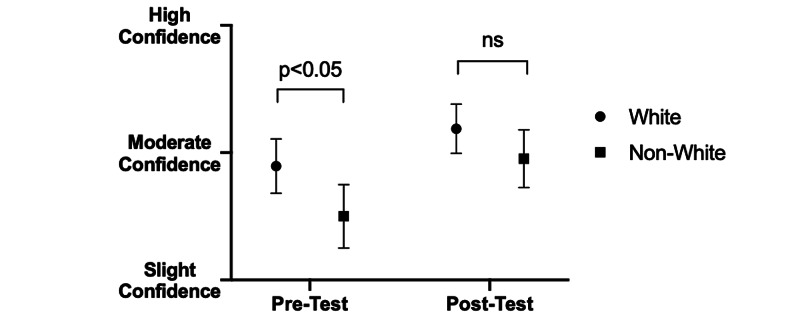
Effect of identifying as white/non-white race on confidence identifying non-verbal cues in patients before and after intervention. White students displayed significantly greater average confidence in recognizing non-verbal cues in patients prior to intervention compared to non-white students (p<0.05). After the intervention, the differences in average confidence among white and non-white students became non-significant, and both groups showed increased average confidence in noticing non-verbal cues among patients, although whites continued to report higher confidence on average than non-white students.

While no initial difference was discernible between students’ self-rated confidence identifying non-verbal cues in other providers based on racial makeup (pre: 2.87 vs. post: 2.50; p>0.05) (Figure 5), white students expressed greater confidence than their non-white peers at identifying such cues in patients before the quartet intervention (pre: 2.90 vs. post: 2.50; p<0.05) (Figure 6). However, this difference was not present afterwards, with no statistically significant difference among white and non-white students in self-expressed confidence identifying non-verbal cues in either healthcare providers (pre: 3.16 vs. post: 2.90; p>0.05) (Figure 5) or patients (pre: 3.19 vs. post: 2.95; p>0.05) (Figure 6). Non-white students exhibited a greater gain in average confidence than white students following intervention (+0.40 vs. +0.26). There was no statistical difference in self-reported confidence with non-verbal cues between self-identified genders either before or after intervention (data not shown).

**Figure 7:**
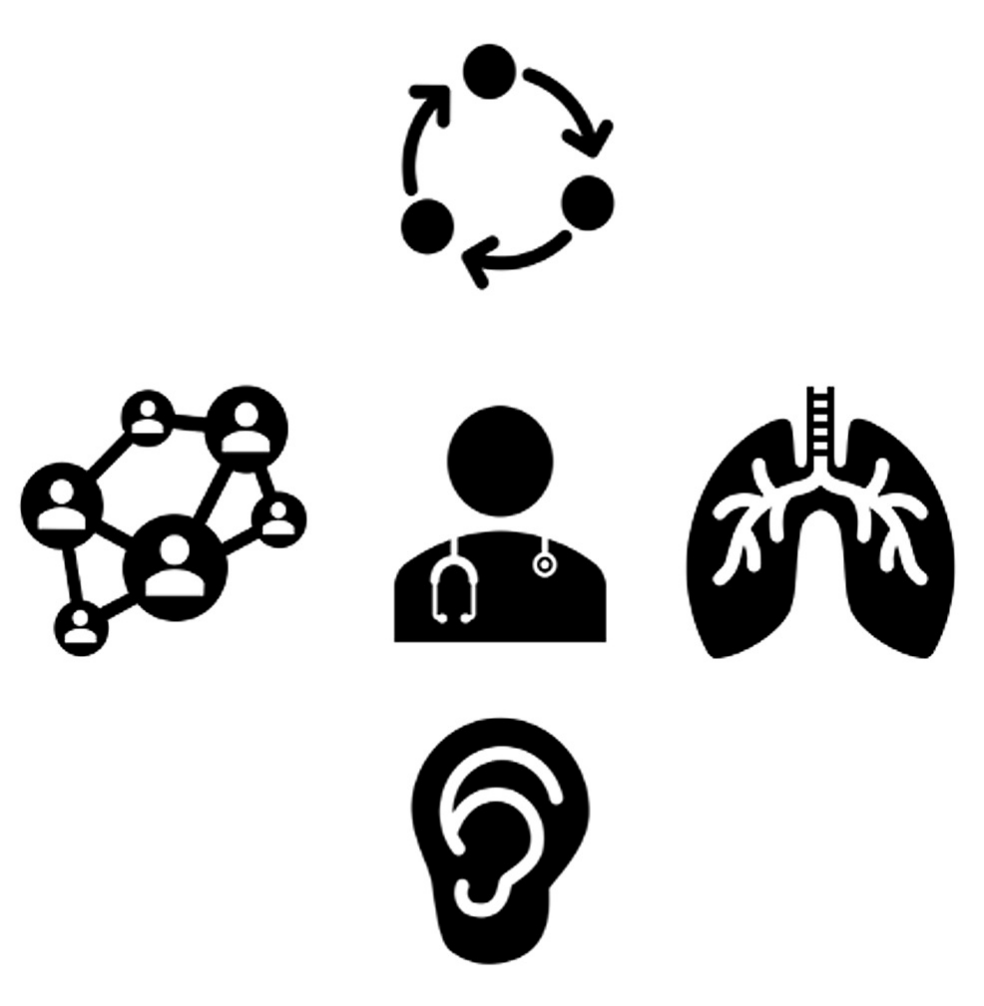
Illustration depictions of communication themes noted in free response among intervention participants. Notable themes regarding non-verbal communication and coordination included (in clockwise fashion) the importance of repetitive team practice (top), the communication value of breath (right), the need for careful listening to things said and unsaid (bottom), and leadership as a team rather than individual endeavor (left).

Notable qualitative themes present before the workshop included (1) leadership as an individual rather than a team endeavor, (2) importance of long-term familiarity among members, and (3) importance of planning a system of non-verbal cues to avoid miscommunication. Themes present after the workshop included (1) importance of iterative team practice, (2) value of breath, (3) need for careful listening, and (4) leadership as a team rather than individual endeavor (Figure 7).

## Discussion

Our results demonstrate that chamber music is an effective vehicle through which third-year medical students are able to gain confidence in recognizing and utilizing non-verbal communication. Our intervention led to global increases in perceived ability to identify non-verbal cues in both patients and healthcare providers. Following the workshop, common non-verbal themes students identified included (1) importance of repetitive team practice, (2) value of breath, (3) need for careful listening among teammates, and (4) leadership as a team rather than individual endeavor.

Previous studies have demonstrated that race plays a significant role with respect to non-verbal communication between patients and medical professionals [8]. LaRochelle and Karpinski [9] showed that racial groups maintain different levels of communication apprehension and that curriculum personnel must account for these factors to maintain students’ communication confidence when working among interprofessional workers. We identified baseline differences in self-reported confidence in identifying non-verbal cues. These differences, however, were mitigated post-intervention, with both white and non-white students exhibiting greater confidence in identifying non-verbal cues without any residual gap between the two groups.

Interestingly, participants with musical backgrounds prior to intervention admitted to significantly lower confidence in identifying non-verbal cues in patients compared with their counterparts without musical backgrounds; following intervention, both groups showed elevated levels of confidence. Due to their previous musical experience, participants with musical backgrounds may have had a greater appreciation for the wide breadth of non-verbal communication utilized by musicians and may paradoxically underestimate their knowledge of non-verbal cues pre-intervention. In contrast, participants without musical experience may have overestimated their understanding of non-verbal cues because they are less familiar with the nuance and intricacy implicit in orchestrating and synchronizing musical ensembles, demonstrating the so-called “Dunning-Kruger” effect [10]. We were happy to see that irrespective of musical background, all participants demonstrated elevations in confidence post-intervention.

Teamwork among medical professionals is linked to improved patient-perceived quality and satisfaction of care [11]. After our brief intervention, student participants recognized discrete themes, e.g., importance of iterative practice, improved listening among team members, and use of breath, all of which have significant overlap in both chamber music and medical teams. In addition, students recognized the importance of iterative practice as a tool to hone non-verbal communication within a team, as compared to a pre-workshop focus on planning a system of agreed-upon non-verbal cues.

Music has been used to teach communication skills to teams. Focusing on the unique role of the orchestral conductor as leader, Larsen et al. [12] developed a course in conjunction with an orchestral conductor to teach team leader-training for emergencies. We explicitly chose chamber musicians (who do not utilize a conductor) to teach non-verbal communication skills because chamber musicians share leadership roles. With increased focus on collaborative team care where healthcare providers share leadership roles, the need to educate medical students with respect to team dynamics, including the vital role of non-verbal communication, is paramount in cultivating a new generation of physician listeners.

There are several limitations in our study. This intervention was performed at a single site and may not be generalizable to other medical schools. We also acknowledge a Hawthorne bias and subject-expectancy effect, given that students from the Internal Medicine and Pediatrics clerkships were required to participate in this workshop. However, we made every effort to communicate that no particular responses were expected and that responses would have no effect on student evaluation for the clerkship. Additionally, we have no definitive clinical measure demonstrating that increased confidence in non-verbal communication translates to improved recognition of non-verbal communication on the clinical wards.

In the future, we plan to follow students who participated in our pilot study to investigate how they believed the workshop impacted their communication skills during subsequent clerkships and rotations. We also plan to compare confidence in non-verbal communication skills between those students who attended the workshop and those who did not to further identify the impact of the session. Future studies are needed to assess the clinical impact of the chamber music workshop on medical students’ non-verbal communication skills.

Careful listening and attention to both verbal and non-verbal cues is vital to patient-centered care. Our intervention highlights that music-based interventions with particular emphasis on the observation and understanding of non-verbal cues can enhance the confidence that medical students express in interpreting the non-verbal cues with patients and other healthcare providers.

## Conclusions

We conclude that quartet intervention leads to a global increase in perceived ability to identify non-verbal cues in both patients and colleagues, while simultaneously erasing pre-existing differences due to musical or racial background. Factors associated with improved confidence in identifying non-verbal cues are (1) musical background and (2) self-identified non-white race. Additionally, we found that common themes students recognized included post-workshop include (1) importance of repetitive team practice, (2) value of breath, (3) need for careful listening, and (4) leadership as a team rather than individual endeavor. Ultimately, observing chamber musicians and guided reflection may improve recognition and future utilization of non-verbal cues among medical students during clinical rotations.

## Figures and Tables

**Figure 8:**
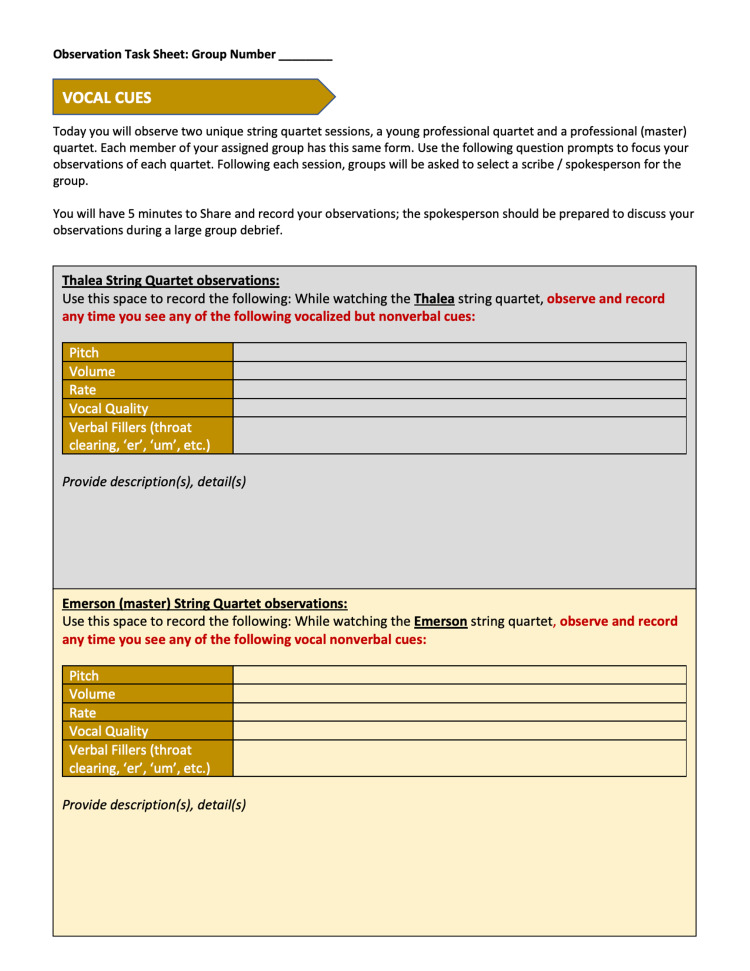
Appendix A

**Figure 9:**
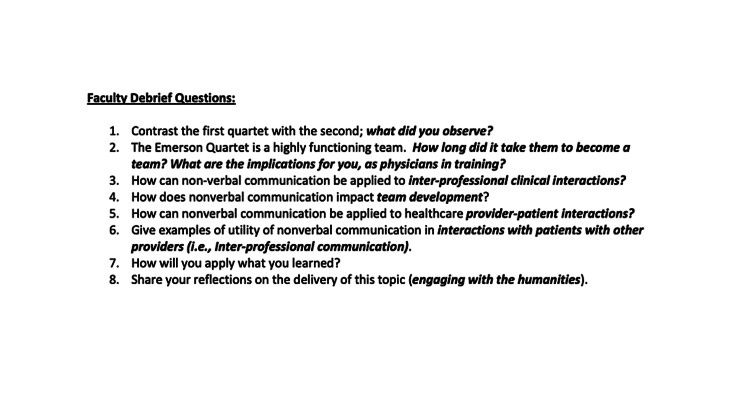
Appendix B

**Figure 10:**
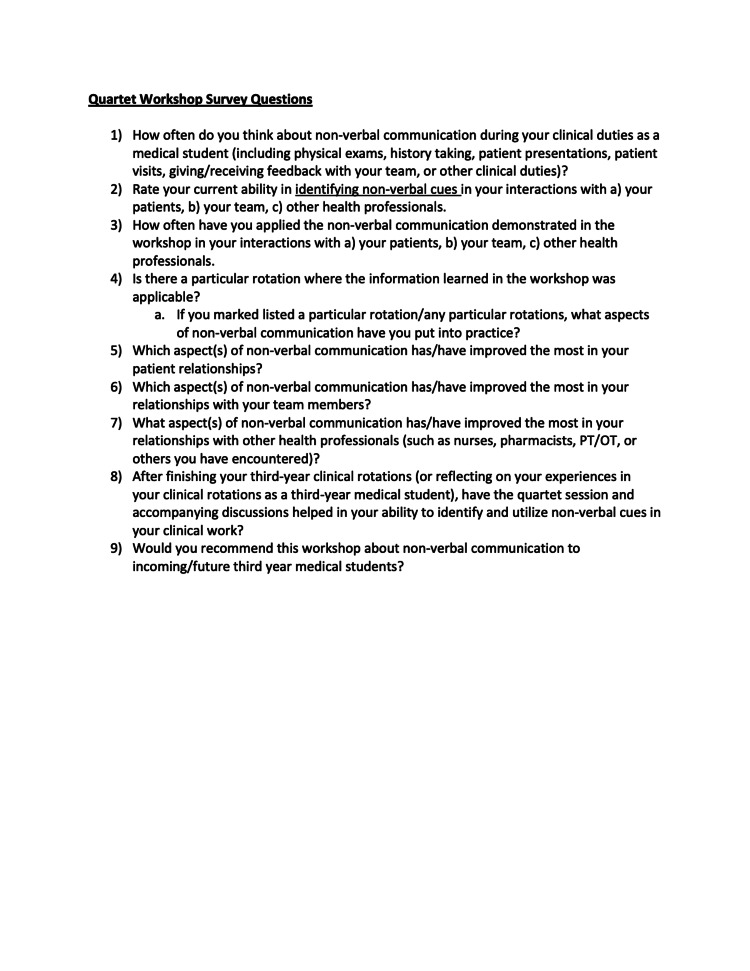
Appendix C
